# Gynecomastia in a Man With Adrenal Mass

**DOI:** 10.1210/jcemcr/luad143

**Published:** 2023-12-20

**Authors:** Jasmine Saini, Patrick Navin, Michael Rivera, Irina Bancos

**Affiliations:** Division of Endocrinology, Metabolism, and Nutrition, Mayo Clinic, Rochester, MN 55905, USA; Division of Radiology, Mayo Clinic, Rochester, MN 55905, USA; Division of Anatomic Pathology, Mayo Clinic, Rochester, MN 55905, USA; Division of Endocrinology, Metabolism, and Nutrition, Mayo Clinic, Rochester, MN 55905, USA

**Keywords:** adrenocortical carcinoma, adrenal cancer, estrogen, feminization

## Abstract

Estrogen-secreting adrenocortical carcinoma (ACC) is exceedingly rare, representing 1% to 2% of all ACCs. We present a case of a 65-year-old man diagnosed with an estrogen-secreting, 4.3-cm right adrenal mass discovered during work-up for bilateral gynecomastia. Gynecomastia and hyperestrogenism resolved after laparoscopic adrenalectomy, and pathology was reported as adrenocortical adenoma. However, 5 years later, he again developed bilateral gynecomastia because of recurrent hyperestrogenism. Imaging revealed multiple metastases in the abdomen. Urine steroid profiling demonstrated increased androgen precursors, androgen metabolites, and glucocorticoid precursors. Ultrasound-guided biopsy of one of the metastases confirmed ACC. Initial therapy included debulking surgery with removal of metastatic lesions. Mitotane therapy was initiated 4 weeks later along with hydrocortisone for anticipated mitotane-induced adrenal insufficiency. Histopathology from the adrenalectomy specimen 5 years earlier was rereviewed and confirmed ACC. Estrogen-secreting adrenal tumors are exceedingly rare, and the majority are malignant. This case underlines the importance of making an initial accurate diagnosis of adrenal malignancy that allows better surgical planning and appropriate monitoring. Indeterminate imaging characteristics of the adrenal mass, as well as the presentation with estrogen excess, suggested an elevated risk for ACC. Initial pathology-based misdiagnosis illustrates the need for an expert adrenal pathologist to review these rare tumors.

## Introduction

Adrenocortical carcinoma (ACC) is a rare and aggressive tumor representing .3% of all adrenal tumors in a population setting and diagnosed at a rate of approximately 1 case per million population (1, 2). Approximately half of all patients with ACC demonstrate either overt or mild hormone excess, most commonly cortisol and/or androgen excess (3). Depending on the degree of hormone overproduction, patients may develop features of overt Cushing syndrome (from excess cortisol), virilization (from excess androgen, evident in women only), or hypertension with hypokalemia (from excess mineralocorticoid). Estrogen-secreting ACCs are very rare and constitute approximately 1% to 2% of ACCs (3, 4). In postmenopausal women, estrogen excess may lead to uterine bleeding, and girls may experience precocious puberty. In men, symptoms of estrogen excess include gynecomastia that occurs from hyperestrogenism-induced breast tissue growth and decreased libido resulting from hypogonadotrophic hypogonadism (4).

We present a rare case of an estrogen-secreting ACC that was initially misdiagnosed as an adenoma, but later presented with metastatic ACC.

## Case Presentation

A 65-year-old man presented to the clinic in June 2022 with bilateral progressive gynecomastia and fatigue. His medical history included dyslipidemia, hypertension, obstructive sleep apnea, gastroesophageal reflux disease, and history of gynecomastia and an adrenal mass 5 years earlier.

**Figure 1.**
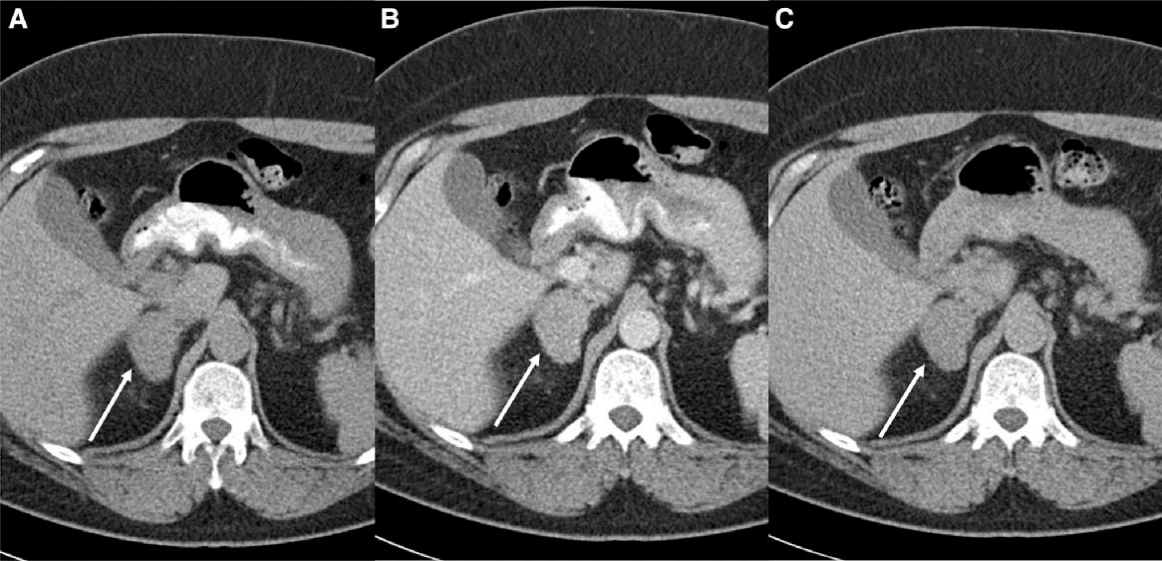
Unenhanced axial CT from 2017 demonstrates a 42-mm right adrenal mass with attenuation of 32 HU (A). Homogenous enhancement was demonstrated on portal venous phase CT (B) with marked washout on delayed 15-minute imaging (C). Absolute contrast washout of adrenal mass is 78% and relative contrast washout is 36%.

**Figure 2.**
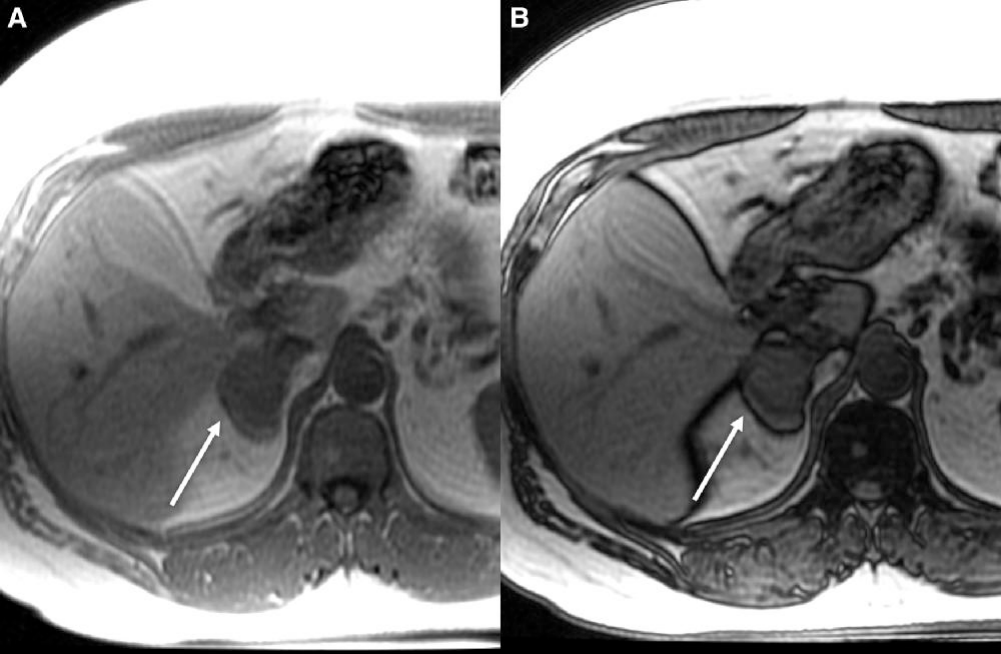
Axial chemical shift MRI demonstrates minimal reduction in signal from in-phase (A) to out-of-phase phase imaging (B), consistent with a lipid-poor indeterminate right adrenal mass.

In 2017, the patient also reported progressive bilateral gynecomastia and fatigue. Laboratory work-up at that time demonstrated elevated estrogen concentrations with the rest of work-up (thyroid function tests, renal and liver function tests) being unremarkable. Testosterone and prolactin measurements were not performed. Finding excess estrogen prompted adrenal imaging. Computed tomography (CT) showed a 42-mm, right adrenal mass with an unenhanced density of 32 Hounsfield units (HU). However, contrast washout characteristics were reassuring, with the absolute washout of 78% and relative washout of 36% (Fig. 1). On the magnetic resonance imaging (MRI), imaging characteristics were indeterminate with absence of chemical shift (Fig. 2).

The patient was treated with a right laparoscopic adrenalectomy locally. Postoperatively, estrogen concentrations decreased 2.5-fold and was within the normal ranges. Two different pathologists assessed histopathology, both concluding that the adrenal mass was benign, consistent with adrenocortical adenoma. The patient was reassured and was not recommended for follow up.

**Figure 3.**
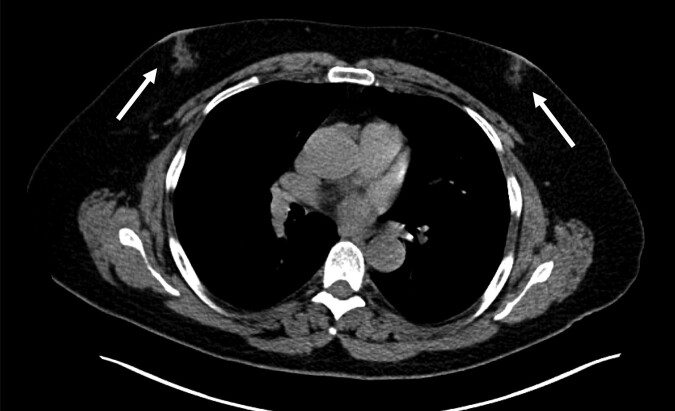
Unenhanced CT through the chest demonstrates bilateral gynecomastia (arrows).

In 2022, after a 5-year symptom-free period, patient redeveloped gynecomastia (Fig. 3) and presented for evaluation to our clinic.

## Diagnostic Assessment

**Figure 4.**
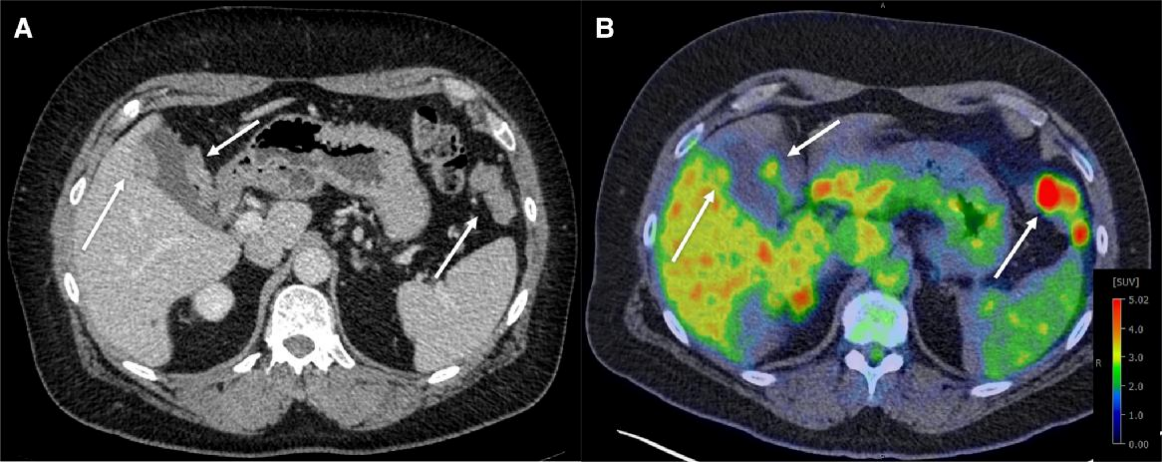
Contrast-enhanced axial CT from 2022 demonstrates enhancing soft-tissue nodules in the left upper quadrant measuring up to 6.7 cm and associated with the gallbladder wall (arrows) and right subdiaphragmatic and periportal nodules A). Axial fused ^18^fluorine fluorodeoxyglucose positron emission tomography-computed tomography demonstrates mild to moderate avidity in the left upper quadrant mass (SUVmax, 9.8) and in the gallbladder wall lesions (SUVmax, 4.2).

**Figure 5.**
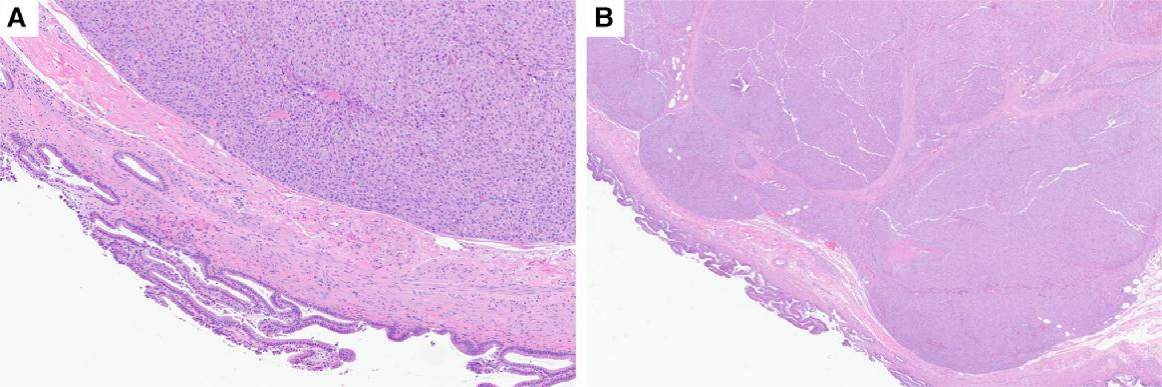
Histology from the gallbladder wall metastasis, hematoxylin and eosin stain shown with original magnification × 4 (A) and × 20 (B) demonstrates that adrenocortical carcinoma undermines the normal appearing gallbladder mucosa. Staining was positive for SF1, Melan A, synaptophysin, and inhibin, and was negative for CK7, CK20, CDX2, and chromogranin.

Considering the patient’s previous history of estrogen-secreting adrenal mass with clinical presentation of gynecomastia, hormonal work-up was repeated, and indeed revealed hyperestrogenism (Table 1). Imaging included abdominal CT, MRI, and positron emission tomography CT (Fig. 4). Urine steroid profiling was consistent with ACC, demonstrating increased estrogen, androgen, and glucocorticoid precursors and metabolites (Z scores of 4 and above) (Table 2). Metastatic ACC was suspected, and the ultrasound-guided left upper quadrant mesenteric mass biopsy confirmed ACC (Fig. 5).

**Table 1.** Laboratory findings

Laboratory findings	Result	Reference range
Estrone	345 pg/mL (1276 pmol/L)	10-60 pg/mL (37-222 pmol/L)
Estradiol	72 pg/mL (264 pmol/L)	10-40 pg/mL (37-147 pmol/L)
Progesterone	0.59 ng/mL (1.9 nmol/L)	<0.20 ng/mL (<0.64 nmol/L)
Total Testosterone	157 ng/dL (5 nmol/L)	240-950 ng/dL (8-33 nmol/L)
Testosterone, bioavailable	16 ng/dL (0.6 nmol/L)	40-168 ng/dL (1.4-5.8 nmol/L)
Cortisol, morning	19 mcg/dL (524 nmol/L)	7-25 mcg/dL (193-670 nmol/L)
Adrenocorticotropic hormone	14 pg/mL (3.08 pmol/L)	7.2-63 pg/mL (1.6-13.8 pmol/L)
Aldosterone	13 ng/dL (0.36 nmol/L)	<=21 ng/dL (<=0.5 nmol/L)
Renin plasma activity	6.3 ng/mL/hr (6.3 mcg/L/hr)	<=0.6-3 ng/mL/hr (<=0.6-3 mcg/L/hr) (Sodium-replete, upright)
11 deoxycortisol	204 ng/dL (5.9 nmol/L)	10-79 ng/dL (0.3-2.3 nmol/L)
Androstenedione	81 ng/dL (2.8 nmol/L)	40-150 ng/dL (1.4-5.2 nmol/L)
Dehydroepiandrosterone Sulphate	219 mcg/dL (5.9 mcmol/L)	12-227 mcg/dL (0.3-6.2 mcmol/L)
Pregnenolone	230 ng/dL (7.3 nmol/L)	33-248 ng/dL (1.04-7.83 nmol/L)
17-Hydroxypregnenolone	264 ng/dL (8.3 nmol/L)	55-455 ng/dL (1.7-14.4 nmol/L)
17-Hydroxyprogesterone	72 ng/dL (2.2 nmol/L)	<220 ng/dL (<6.6 nmol/L)

**Table 2.** 24-hour urine steroid profiling

Steroid	Before debulking surgery		2 mo after surgery		Reference ranges (mcg/24 h)
	Value (mcg/24 h)	Z score	Value (mcg/24 h)	Z score	
Androsterone	1986	0	609	−1.2	118-25389
Etiocholanolone	3135	1	1141	−.3	127-15640
Dehydroepiandrosterone	12 595	4	298	.5	7-4260
16a-OH-dehydroandristepiandrosterone	3687	2	1361	1.6	11-6183
5-Pregnenetriol	14 475	6	480	1.0	24-2162
5-Pregnenediol	4023	5	150	.0	17-1296
Tetrahydro-11-corticosterone	637	2	145	−.1	16-1674
Tetrahydro-11-deoxycorticosterone	72	2	Undetectable	—	5-297
Pregnanediol	4336	4	165	−.3	23-1846
17a-OH-Pregnanolone	458	1	113	−.6	18-1747
Pregnanetriol	4952	3	324	−1.4	115-5432
Pregnanetriolone	29.2	0	Undetectable	—	5-221
Tetrahydrodeoxycortisol	3343	4	102	−.2	12-1277
Cortisol	228	2	149	.9	12-597
6B-OH-Cortisol	483	1	1552	2.4	22-2406
Tetrahydrocortisol	4230	1	4375	.8	331-19009
5a-Tetrahydrocortisol	3676	1	1406	−.6	155-35266
B-Cortol	1675	2	207	−1.1	56-3541
11B-OH-Androsterone	1164	0	763	−.8	142-13135
11B-OH-Etiocholanolone	1114	1	1568	1.1	69-6805
Cortisone	225	1	123	−.2	24-732
Tetrahydrocortisone	10 650	1	6724	.7	454-34576
a-Cortolone	3412	1	2872	.5	211-17591
B-Cortolone	2024	1	580	−.7	114-8434
11-Oxoetiocholanolone	467	−1	657	−.7	155-7174

**Figure 6.**
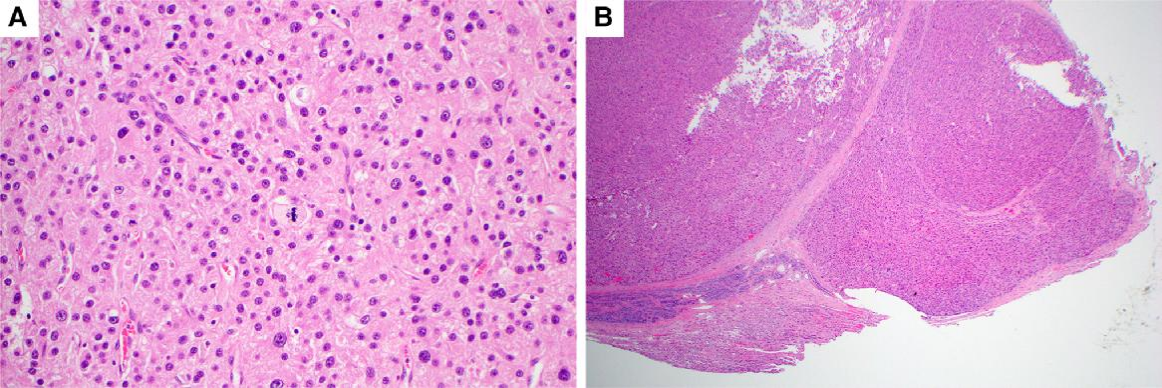
Histology from the adrenal mass removed in 2017, hematoxylin and eosin stain shown with original magnification × 4 (A) and × 20 (B) demonstrates irregular nodular growth pattern consistent with capsular invasion and enlarged pleomorphic hyperchromatic nuclei and mitotic activity.

Considering the patient’s previous diagnosis of benign adrenal adenoma and current presentation with ACC, pathology slides from the previous surgery were requested and reviewed by an expert adrenal pathologist. This review confirmed that the initial adrenal mass from 2017 was misinterpreted as benign and was in fact ACC, Fig. 6, reporting foci suspicious for capsular invasion, marked cytological atypia, diffuse architecture, < 25% clear cells, and increased mitoses of 23/50 high power fields, with a Weiss score of 4 of 9.

## Treatment

Initial therapy included debulking surgery with the removal of multiple metastatic lesions.

Postoperatively, estrogen (13 pg/mL normal ranges, 10-40 pg/mL [48 pmol/L]) and urine steroid profiling (Table 2) normalized, and gynecomastia improved when reevaluated 3 months later.

## Outcome and Follow-up

Imaging surveillance was initiated and planned for every 3 months during the first 12 months after adrenalectomy. Mitotane therapy was initiated 4 weeks after adrenalectomy along with hydrocortisone for anticipated mitotane-induced adrenal insufficiency. When imaging demonstrated progressive disease at 3 the month-follow up visit, cytotoxic chemotherapy with etoposide, doxorubicin, and cisplatin was initiated, and mitotane was discontinued in an attempt to reduce the overall toxicity. Following a favorable response to the 6 cycles of etoposide, doxorubicin, and cisplatin, our patient demonstrated a favorable response, and at this time is under observation without further therapy. CT performed 8 months after chemotherapy initiation demonstrated no metastases except for 1 peritoneal nodule that decreased in size when compared with previous imaging.

## Discussion

We describe a patient with an estrogen-secreting adrenal mass that was initially misdiagnosed as having an adrenal adenoma and who developed recurrent estrogen excess because of metastatic ACC 5 years after adrenalectomy. At the time of initial presentation, the patient’s imaging characteristics of adrenal mass were indeterminate and included tumor size >4 cm, unenhanced HU >20, and absence of chemical shift on MRI. However, the patient had reassuring contrast washout characteristics with absolute washout >60% (though a relative washout of 36%).

Making an accurate diagnosis of ACC is important to assure appropriate management. Imaging characteristics that are concerning for malignancy include unenhanced HU > 20 (or heterogenous mass) and size > 4 cm (5). Contrast washout is not recommended as a second-line imaging because of scarce data and suboptimal performance (5). Combined hormone excess, androgen, or estrogen excess in a patient with indeterminate adrenal mass also strongly suggests ACC (5). Because approximately 50% of ACCs do not demonstrate abnormalities on standard of care hormonal work-up, urine steroid profiling can aid the diagnosis of ACC (5).

Data on estrogen-secreting adrenal tumors are scarce. A literature review of 21 cases of estrogen-secreting adrenal tumors in men published between 1970 and 2014 reported that 71% were malignant, and 2 cases of ACC were initially misclassified as adenoma, similar to our case (4). Since 2014, 5 additional cases of estrogen-secreting tumors in men were published, with 3 of 4 with optimal follow-up being malignant, and 1 case of reportedly benign adenoma that was followed for 6 months only (6-9).

Histopathology is considered the gold standard for diagnosing ACC. Guidelines recommend review of all suspected ACC specimens by an expert adrenal pathologist because of the frequent risk of misdiagnosis: 26 (9%) of cases were misdiagnosed in 1 study of 300 patients with ACC and in another study of 161 patients with ACC, initial diagnosis was revised by reference pathologist in 21 (13%) patients (10). Weiss score is a histological criterion of 9 parameters (nuclear grade, mitosis per 50 high-power field, atypical mitotic figure, % of clear cells, diffuse architecture, necrosis, vascular invasion, sinusoidal invasion, capsular invasion) that can help differentiate malignant from benign adrenal tumors. The score is calculated on hematoxylin and eosin-stained slides, and score of 3 or greater indicates ACC. Ki67 immunohistochemistry index can help make a diagnosis of ACCs as well as stratify the prognosis of ACC and guide management. Our patient's resected histopathology specimen was labeled as benign in 2017. Five years later, when the patient presented with recurrent hyperestrogenism, the initial histopathology was reviewed by an adrenal expert pathologist who confirmed ACC with a Weiss score of 4/9.

Although open adrenalectomy is recommended for an adrenal mass suspected to be ACC, minimally invasive adrenalectomy could be considered by an expert high-volume adrenal surgeon in ACCs < 6 cm without evidence of local invasion. The recommended standard of care approach to localized ACC is imaging follow up for at least 5 years after adrenalectomy and mitotane therapy if the patient is at substantial risk of recurrence (10).

## Learning Points

- Estrogen-secreting adrenocortical carcinomas (ACCs) represent 1% to 2% of all ACCs.
- Establishing an accurate adrenal malignancy diagnosis before adrenalectomy is important and includes review of imaging characteristics (size, Hounsfield Unit, homogeneity) and interpretation of hormonal work-up.
- The resected specimen in suspected ACC cases should be reviewed by an expert adrenal pathologist.

## Contributors

All authors made individual contributions to authorship. J.S. participated in drafting the manuscript. P.N., M.R., and I.B. participated in the diagnosis and management of this patient and drafting of the manuscript. M.R. undertook histopathology section and preparation of histology images. P.N. undertook radiological imaging and preparation of radiology reports. All authors critically reviewed and approved the final draft.

## Data Availability

Data sharing is not applicable to this article as no datasets were generated or analyzed during the current study.

## Abbreviations

ACC: adrenocortical carcinoma
CT: computed tomography
HU: Hounsfield unit
MRI: magnetic resonance imaging
